# The de.NBI / ELIXIR-DE training platform - Bioinformatics training in Germany and across Europe within ELIXIR

**DOI:** 10.12688/f1000research.20244.2

**Published:** 2020-09-11

**Authors:** Daniel Wibberg, Bérénice Batut, Peter Belmann, Jochen Blom, Frank Oliver Glöckner, Björn Grüning, Nils Hoffmann, Nils Kleinbölting, René Rahn, Maja Rey, Uwe Scholz, Malvika Sharan, Andreas Tauch, Ulrike Trojahn, Björn Usadel, Oliver Kohlbacher

**Affiliations:** 1 Center for Biotechnology (CeBiTec), Bielefeld University, Bielefeld, 33501, Germany; 2 Bioinformatics Group, Department of Computer Science, Albert-Ludwigs-University Freiburg, Freiburg, 79110, Germany; 3 Bioinformatics and Systems Biology, Justus-Liebig-University Giessen, Giessen, 35392, Germany; 4 Alfred-Wegener-Institut - Helmholtz Zentrum für Polar- und Meeresforschung and Jacobs University Bremen, Campus Ring 1, Bremen, 28759, Germany; 5 Leibniz-Institut für Analytische Wissenschaften – ISAS – e.V., Dortmund, 44227, Germany; 6 Algorithmic Bioinformatics, Department of Mathematics and Computer Science, Freie Universität Berlin, Takustraße 9, Berlin, 14195, Germany; 7 Scientific Databases and Visualization Group, Heidelberg Institute for Theoretical Studies (HITS) gGmbH, Schloss-Wolfsbrunnenweg 35, Heidelberg, 69118, Germany; 8 Leibniz Institute of Plant Genetics and Crop Plant Research (IPK) Gatersleben, Seeland, 06466, Germany; 9 The Heidelberg Center for Human Bioinformatics (HD-HuB), European Molecular Biology Laboratory, Meyerhofstrasse 1, Heidelberg, 69117, Germany; 10 IBG-2 Plant Sciences, Forschungszentrum Jülich, Jülich, 52428, Germany; 11 Applied Bioinformatics, Department of Computer Science, University of Tübingen, Tübingen, 72076, Germany; 12 Institute for Bioinformatics and Medical Informatics, University of Tübingen, Tübingen, 72076, Germany; 13 Translational Bioinformatics, University Hospital Tubingen, Tübingen, 72076, Germany; 14 Biomolecular Interactions, Max Planck Institute for Development Biology, Tübingen, 72076, Germany

**Keywords:** de.NBI, de.NBI Cloud, Life Sciences, Bioinformatics, ELIXIR, Training, Education, Germany

## Abstract

The German Network for Bioinformatics Infrastructure (de.NBI) is a national and academic infrastructure funded by the German Federal Ministry of Education and Research (BMBF). The de.NBI provides (i) service, (ii) training, and (iii) cloud computing to users in life sciences research and biomedicine in Germany and Europe and (iv) fosters the cooperation of the German bioinformatics community with international network structures. The de.NBI members also run the German node (ELIXIR-DE) within the European ELIXIR infrastructure. The de.NBI / ELIXIR-DE training platform, also known as special interest group 3 (SIG 3) ‘Training & Education’, coordinates the bioinformatics training of de.NBI and the German ELIXIR node. The network provides a high-quality, coherent, timely, and impactful training program across its eight service centers. Life scientists learn how to handle and analyze biological big data more effectively by applying tools, standards and compute services provided by de.NBI. Since 2015, more than 300 training courses were carried out with about 6,000 participants and these courses received recommendation rates of almost 90% (status as of July 2020). In addition to face-to-face training courses, online training was introduced on the de.NBI website in 2016 and guidelines for the preparation of e-learning material were established in 2018. In 2016, ELIXIR-DE joined the ELIXIR training platform. Here, the de.NBI / ELIXIR-DE training platform collaborates with ELIXIR in training activities, advertising training courses via TeSS and discussions on the exchange of data for training events essential for quality assessment on both the technical and administrative levels. The de.NBI training program trained thousands of scientists from Germany and beyond in many different areas of bioinformatics.

## Introduction

In the last decade, researchers in the life sciences have been early victims of the ‘Big Data Problem’ because of technical improvements in the so-called ‘omics’ and image analysis fields including the challenges of the five ‘V’s of big data: volume, veracity, velocity, variety, and value ^1^. Here, large and complex datasets are rapidly generated to analyze various biological levels in living cells per day. Even small benchtop sequencing machines are now capable of producing terabytes of data, but many life scientists neither possess the skills to analyze the data properly nor the knowledge to use existing analysis resources. Therefore, the true bottleneck of the current ‘Big Data Problem’ in the life sciences is often not the storage or compute power, but the knowledge and skills how to use existing bioinformatics services and tools. An important way to solve these deficiencies in the field of life sciences is by means of bioinformatics training. The need for such training was described recently: The majority (> 95%) of life scientists located in Europe work or plan to work with large datasets, but less than 35% possess the bioinformatics and statistical skills to handle the huge amount of generated data ^2^.

### Introduction to the German Network for Bioinformatics Infrastructure (de.NBI)

In 2012, the German Bioeconomy Council published the recommendations ‘Requirements for a Bioinformatics Infrastructure in Germany for future Research with bioeconomic Relevance’, coming to the conclusion that expertise centers in Germany should have permanent structures for training users from life sciences ^3^.

**Figure 1.**
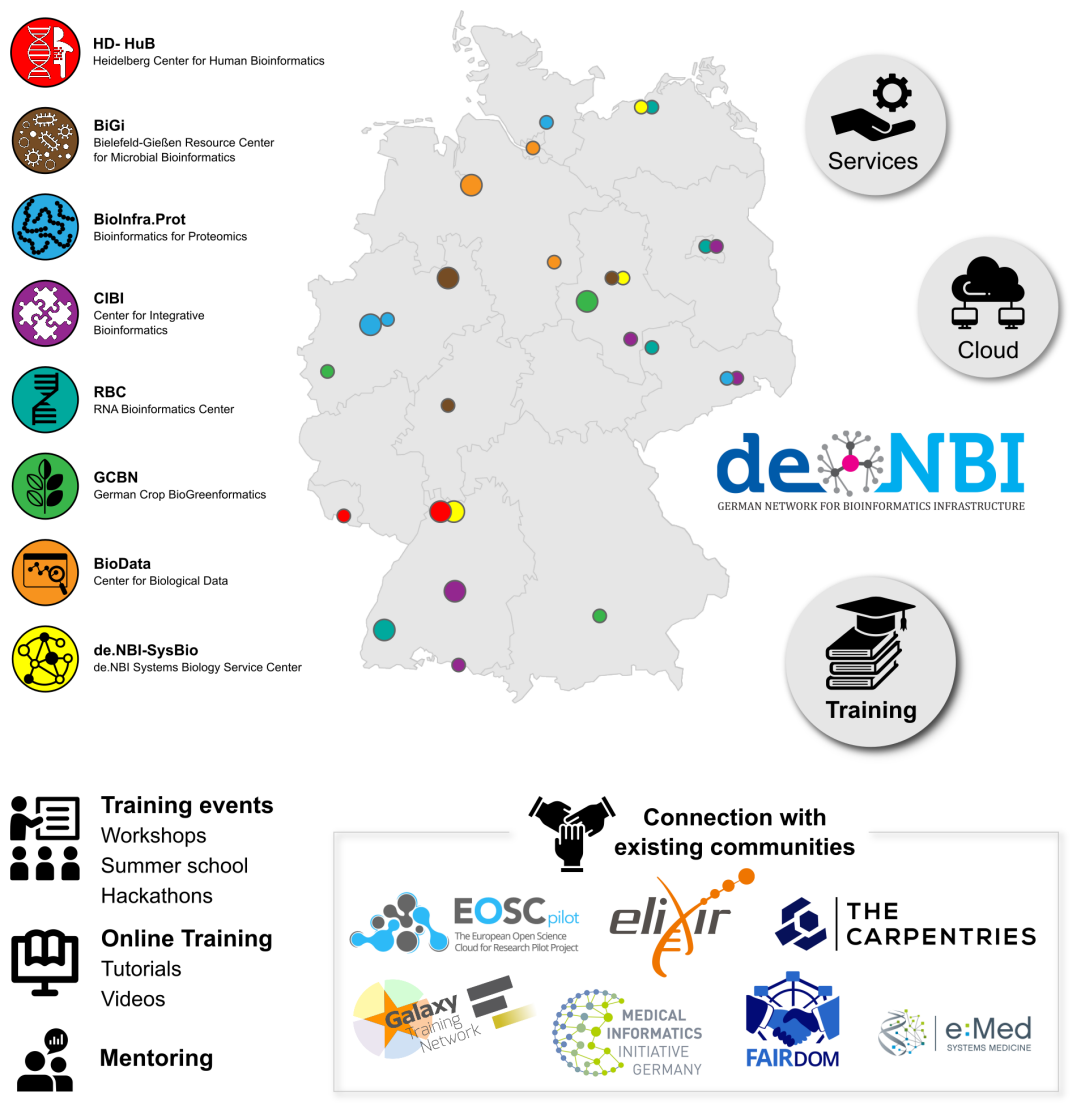
Training topics and locations of the eight de.NBI service centers in Germany and their connections with existing German and European communities. On the left side, the service centers are shown. These centers organize specific training events, create online training material and provide mentoring. In the middle, the map of Germany includes all de.NBI locations. The different colors represent the corresponding service center (see logos on the left side).

Accordingly, the German Network for Bioinformatics Infrastructure (de.NBI) program was launched by the Federal Ministry of Education and Research (BMBF) in March 2015, and it now includes 40 projects that are operated by 30 research institutes organized in eight service centers ^3^. de.NBI offers a large repertoire of high-quality training courses to support life scientists with different expertise levels in bioinformatics and from various research fields. These training activities of de.NBI are focused on teaching life scientists in Germany and Europe the proper handling and analysis of their biological big data more effectively by applying tools, standards and compute services provided by the service centers. Therefore, de.NBI trainers work together and within different existing German and European (training) communities, e.g. the Galaxy Training Network ^2^, The Carpentries ^4^, FAIRDOM ^5^ etc., to connect de.NBI to researchers and to the most important topics in the life sciences community ( Figure 1). These topics were introduced in the de.NBI training activities, which range from basic skills to advanced data analysis and expert hackathons including 1–14 day training courses, webinars, mentoring, online training and one-week summer schools ^3^. All these training activities are listed on the de.NBI webpage and on TeSS. TeSS is ELIXIR's training platform, providing a one-stop shop for trainers and trainees to discover online information and content, including training materials, events and interactive tutorials.

### Introduction to ELIXIR-DE

Since August 2016, Germany is member of the European life-sciences infrastructure for biological information, ELIXIR. The Federal Ministry of Education and Research (BMBF) signed the ELIXIR Consortium Agreement establishing Germany as member no. 20 of the distributed infrastructure that unites Europe’s leading life science organizations. The German Network for Bioinformatics Infrastructure (de.NBI) has been designated by the BMBF to establish the German node in ELIXIR by providing high quality bioinformatics services and training expertise to the scientific user community in Europe. 

On 9 January 2020, the National Collaboration Agreement to establish the German ELIXIR Node (ELIXIR-DE) became effective. This contract was signed by 21 de.NBI members (German universities and research institutes), including Bielefeld University that will take over the legal role as ‘Representing Entity‘ of ELIXIR Germany. The National Collaboration Agreement is the legal document that specifies the organization of ELIXIR-DE and the role of the Central Coordination Unit (CCU).

In addition, the ELIXIR Collaboration Agreement was signed by the Representing Entity and the ELIXIR Director, Niklas Blomberg. This contract became effective on 10 February 2020, thereby fully establishing the German ELIXIR Node. The ELIXIR Collaboration Agreement is the legal document that ties together the ELIXIR Hub with the national node and it forms the legal basis of Commissioned Services. These technical projects guide future service developments.

Although not being part of the German ELIXIR Node, EMBL Heidelberg is a consortium partner in de.NBI and is a strong collaboration partner and contributor of the German ELIXIR Node through its own ELIXIR Node (Node to Node collaboration). The list of services provided by EMBL to the German Node has been included in the EMBL ELIXIR Work Programme, which has been approved by the ELIXIR Board in April 2019. The EMBL ELIXIR Work Programme therefore represents the legal basis of Commissioned Services carried out at EMBL.

### Introduction to the de.NBI / ELIXIR-DE training platform

The special interest group 3 (SIG 3) ‘Training & Education’ is centrally coordinating the training courses and the education efforts within de.NBI and ELIXIR-DE. It is composed of 19 training experts from each service center and is led by Oliver Kohlbacher (Eberhard Karls University of Tübingen - Chair) and Daniel Wibberg (Bielefeld University - Deputy Chair) (status as of July 2020). SIG 3 collects all planned de.NBI training activities and provides a structured process of reporting, documenting, and monitoring all training events. The general goal of de.NBI is to develop a national bioinformatics training program for Germany.

The following topics are currently in the focus of SIG 3:

- Improvement of the quantity and quality of training courses.
- Introduction of online training/e-learning courses/platforms.
- Standardization of training course monitoring.
- Integration into the ELIXIR training platform and TeSS.
- Coordination of training events and standards with ELIXIR.

The strategic planning of SIG 3 includes following aspects:

- Closing of gaps in the topics covered by the training courses.
- Improvement of the quantity and quality of e-learning material.
- Qualification of more trainers, e.g. by more Train-the-Trainer courses and Trainer meeting.

The training activities of each de.NBI service center are described in the following sections.

## Bioinformatics training in Germany by de.NBI

### Heidelberg Center for Human Bioinformatics (HD-HuB)

The Heidelberg Center for Human Bioinformatics (HD-HuB) unites bioinformatics expertise from research groups at four established research institutions in Heidelberg and Berlin: European Molecular Biology Laboratory (EMBL), the German Cancer Research Center (DKFZ), Heidelberg University and Berlin Institute of Health (BIH) at Charité. Since November 2016, a partner project on computational epigenetics (de.NBI-Epi) has been added to HD-HuB, which is coordinated by the researchers of Saarland University in association with DKFZ.

***Research focus.*** HD-HuB service center focuses on delivering computational resources to support the development and maintenance of bioinformatics tools in four main application areas within the organizational framework of de.NBI:

1. High-throughput sequencing (HTS) data analysis to study human genetics and genomics ^6^.
2. Taxonomic and functional microbiome profiling, genome and metagenome annotation and association of microbiome composition with host phenotypes to facilitate metagenomic studies ^7^.
3. Automated analysis and visualization of large-scale high-content phenotype screening data for phenotyping of human cells ^8, 9^.
4. Analysis workflows for genome wide DNA methylation data for computational epigenetics research ^10^.

In addition, HD-HuB members established two of the six de.NBI Cloud sites in Heidelberg and Berlin.

***Training and outreach activities.*** The HD-HuB consortium has offered training courses addressing the computational needs of early stage and advanced researchers with a wide variety of research interests. The de.NBI Cloud compute resources are used in different HD-HuB training activities. Below, HD-HuB’s training activities are listed under different categories based on the different learner-profiles:

1. **Programming courses for novice and advanced learners.** HD-HuB members from EMBL organize several beginner and advanced courses on programming skills every year in Python, R, Unix/Shell and version control. These courses have been positively received by the course participants, who constitute ~50% of our overall learners every year. Such courses have been vital to the computational skill development of biologists and wet-lab scientists, who lack formal training in computation and bioinformatics. In addition, The Carpentries courses have been very popular among the novice learners in our communities that allow them to acquire the basic building blocks of software development at the *Software Carpentry* workshops and get hands-on experience of data analysis at the *Data Carpentry* workshops.
2. **Training on specialized bioinformatics topics**. In addition to offering general training courses, the HD-HuB service center offers specialized courses as per demand from the researchers with different research backgrounds. These so far include courses on protein bioinformatics, bioimage analysis, DNA methylation, experimental data analysis and RNA-Seq and Bisulfite sequencing data analysis in cancer research.
3. **Courses for advanced computational biologists.** To support computational needs of advanced learners in our community, we offer hands-on training on data analysis skills such as machine learning, workflow management, cloud computing and statistics. These workshops also provide an opportunity for peer-based learning by bringing scientists with similar research interests together who can potentially collaborate with each other in the future.
4. **Train the trainer courses.** Trainers at our workshops are often volunteers from the respective scientific community, who spend time in creating useful resources to teach at these events. These volunteers are highly valuable to our mission of training computational skills to the future learners. In order to facilitate such knowledge transfer effectively, HD-HuB has started to train experienced researchers, who can become trainers in future courses at their respective institutes. These trainings are offered under the title ‘train the trainer’ where they can gain theoretical and practical knowledge of useful teaching techniques used in best teaching practices.
5. **Local outreach activities.** HD-HuB hosted the first international de.NBI symposium ‘Bioinformatics for Human Health and Disease’ in 2016 at the German Cancer Research Center (DKFZ) in Heidelberg. Attended by 140 participants and over 20 international speakers, the conference provided an opportunity for the researchers to discuss their research and plans within the de.NBI framework. HD-HuB has also co-hosted local event series like de.NBier technical seminars and the Heidelberg Unseminar in Bioinformatics, which are mainly targeted at non-expert scientific communities.

### Bielefeld-Gießen Resource Center for Microbial Bioinformatics (BiGi)

BiGi consists of three partners: the Genome Informatics group at Bielefeld University, the Bioinformatics & Systems Biology group at Justus-Liebig-University Gießen and the partner project at Otto-von-Guericke-University Magdeburg.

***Research focus.*** The BiGi service center offers tools, services and training for microbial genome ^11– 14^, metagenome ^15^, and postgenome research ^16^ that is complemented by a large-scale hardware infrastructure. The focus in Gießen is on genomics (assembly, annotation and comparative analysis and short-read-mapping data evaluation) and read-based metagenomics research. Bielefeld focuses on assembly-based metagenomics and post-genome analysis, such as transcriptomics, proteomics and metabolomics. The partner project, located in Magdeburg, develops solutions for the analysis of metaproteome data. The BiGi service center concentrates on the computational analysis of isolated microbes as well as of microbial communities. It is equipped with large-scale computing and storage resources, and the institutes in Bielefeld and Gießen were involved in establishing a dedicated de.NBI Cloud infrastructure.

Since the establishment of the de.NBI Cloud ^17, 18^, all partners work on different cloud-based applications such as tailor-made images for specific bioinformatics analysis (Metagenomics, Nanopore sequencing, ASA ^3^P ^19^) that also play a key role in many of the workshops provided.

***Training and outreach activities.*** Researchers, who are interested in the analysis of genomic, metagenomic or postgenomic data or who want to get familiar with the use of the de.NBI Cloud ^17, 18^ are welcome to join one of the workshops offered by BiGi.

1. **Nanopore best practice workshop.** To keep up with recent developments in sequencing techniques, a Nanopore best practice workshop has been launched in Bielefeld in 2017 with the support of the service center GCBN that will be held annually due to high demand of the scientific community. The aim of this workshop is to familiarize the participants with the Nanopore sequencing technology, its applications and the ‘Best Practice’ bioinformatics workflow. The Nanopore technology has greatly facilitated the assembly of prokaryotic and eukaryotic genomes. Therefore, the workshop concentrates on the establishment of finalized genome sequences.
2. **Training on genomics tools.** Since the start of the de.NBI network, Gießen offers an annual genome analysis workshop. Topic of this three-day-workshop is microbial sequence data analysis including quality control, assembly, genome annotation and comparative genomics with a focus on the usage of the BiGi software tools ASA ^3^P ^19^ and EDGAR ^12– 14^ as well as the BiGi Galaxy Server.
3. **Training on metagenomics tools.** A further annual course organized by Bielefeld and Gießen together is the introduction into targeted and untargeted metagenome analysis. This course teaches best practices for targeted (16S rDNA operon gene amplicons) as well as untargeted (whole-genome shotgun) metagenome analysis based on high throughput next-generation sequencing data.
4. **Training on postgenomics tools.** For post-genomic analyses, there is a workshop on the analysis, visualization and integration of multi-level -omics data, which introduces the web-based platform Omics-Fusion ^20^. Aim of this workshop is to give answers to typical questions in omics data analyses, e.g. regarding data normalization strategies and handling of missing values, the detection of groups of transcripts, proteins and/or metabolites with similar patterns of expression/abundance using cluster analyses, the visualization of multi-omics data in the context of metabolic pathways, or the identification of differentially regulated transcripts, proteins, and metabolites.
5. **Training on metaproteomics tools.** A workshop on the MetaProteomeAnalyzer ^21^, also including a wet-lab part in some courses, is provided at least once a year in Magdeburg. The learning goal is to apply a complete workflow starting from design of experiments via sample preparation to measurement and bioinformatics analysis of high-resolution mass spectrometry (MS) data.
6. **Introduction to the de.NBI Cloud.** There are basic courses on the use of the de.NBI Cloud ^17, 18^, which are offered in Bielefeld and Gießen or also as dedicated workshops on scientific conferences. In these courses, participants learn to setup a research project on the de.NBI Cloud, to work with virtual instances, to efficiently utilize cloud computing resources, about networking and security issues and means of deploying bioinformatics tools in the cloud.

### Bioinformatics for Proteomics (BioInfra.Prot)

The Bioinformatics for Proteomics (BioInfra.Prot) service center is located within the medical bioinformatics group at the Medizinisches Proteom-Center at Ruhr-University Bochum and at the Leibniz-Institut für Analytische Wissenschaften - ISAS - e.V. in Dortmund. Within the Lipidomics Informatics for Life Sciences (LIFS) partner project of the center, three members are involved: the Biological Mass Spectrometry group at the Max Planck Institute of Molecular Cell Biology and Genetics (MPI-CBG) in Dresden, the bioanalytical chemistry group at Forschungszentrum Borstel - Leibniz Lungenzentrum, and the lipidomics group at ISAS in Dortmund.

***Research focus.*** The main topic of BioInfra.Prot is proteomics data standardization and conversion, protein inference, quality standards, expression analysis and bioinformatics and statistical consulting ^22^. BioInfra.Prot develops and maintains tools such as PIA ^23^ for protein inference and identification, PAA ^24^ for biomarker detection from protein microarray experiments, and SearchGUI ^25, 26^ and PeptideShaker ^26, 27^ for combined scoring of proteins from multiple search engines to enable automated high-throughput processing of large-scale proteomics data from clinical and general life sciences contexts.

The LIFS partner project mainly works on bioinformatics for targeted and comparative lipidomics with applications in cardiovascular and platelet-related disease characterization including lipid identification and discovery ^28^. A further focus is placed on lipidomics data standardization and the establishing of a lipid-centric repository for high-resolution mass spectrometry data. LIFS develops and maintains the tools LipidXplorer ^29^ for shotgun and LipidCreator for LC-MS lipidomics, as well as LipidCompass as a quantitative reference database for tissue and organism-specific lipidomes, and LUX Score ^30^ for comparative visualization of lipidomes based on their shared lipid structural space.

***Training and outreach activities.*** BioInfra.Prot offers courses mainly focused on mass-spectrometric data analysis and integration in proteomics and lipidomics. Therefore, the courses are mainly aimed either at researchers coming from life or analytical science and working with mass spectrometry data, who want to learn the basic tools and workflows of data preprocessing, statistical analysis and integration, or at bioinformaticians, who want to extend their knowledge on available tools and workflows in these areas.

1. **Training on proteomics tools.** BioInfra.Prot offers regular courses on bioinformatics for proteomics at the annual German Society for Mass Spectrometry (DGMS) meeting and at Ruhr-University Bochum. These courses are generally open to anyone, providing material for beginners and more advanced participants alike. The online announcement of each course includes information on recommended prerequisites.
2. **Training on biostatistics / statistical analysis.** BioInfra.Prot also offers trainings for differential analysis of quantitative proteomics data and for general introductions to biostatistics and statistical analysis with R. These courses also target beginners and more advanced participants. New courses are regularly announced *via* the de.NBI portal and on the BioInfra.Prot website.
3. **Training on lipidomics tools.** LIFS offers an annual course for lipidomics bioinformatics tools at the Lipidomics Forum conference (ISAS Dortmund and FZ Borstel). This course is generally targeted at beginners and intermediate users with wet lab or bioinformatics background who want to learn about the analytical and bioinformatics challenges in lipidomics, how to apply the tools developed by LIFS within a lipidomics workflow, and which data formats to use for reporting of their results.
4. **Winter and summer schools.** Both BioInfra.Prot and LIFS have (co-)organized summer schools, e.g. the de.NBI Summer School 2016 - From Big Data to Big Insights, the EUBIC winter schools 2017 and 2019 and the e:Med LipoSysMed summer school 2019. These events help to connect and deepen the interaction between the biological, analytical chemistry, clinical medicine and bioinformatics communities by providing more in-depth, audience-specific hands-on tool trainings.

### Center for Integrative Bioinformatics (CIBI)

The Center for Integrative Bioinformatics (CIBI) joins the three projects OpenMS ^31^, SeqAn ^32^ and KNIME ^33^ that are developed and maintained by researchers at three well established research institutes within Germany: Eberhard Karls University of Tübingen, Free University of Berlin and the University of Konstanz. In addition, the two partner projects MASH ( Leibniz Institute of Plant Biochemistry, Halle/Saale) and DAIS ( Max Planck Institute for Molecular Cell Biology and Genetics, Dresden) joined CIBI in November 2016.

***Research focus.*** CIBI delivers cutting-edge software solutions covering a large domain in the field of computational biology, biomedicine and bioimaging as well as their integration into the workflow system KNIME Analytics platform ^33^ to enable data-driven innovations and achievements in these fields. Concrete, the service center develops state-of-the-art and strongly competitive tools in the fields of proteomics and metabolomics, genomics, image processing, data mining and workflow integration, with the goal of reducing time and cost expenses for various bioinformatics and biomedical data analysis tasks.

***Training and outreach activities.*** Within CIBI, a broad spectrum of training activities is offered to cover on the one hand the large domain the service center is actively contributing to and secondly to offer distinct training events for different target groups, such as data scientists dealing with big omics-data or application developers and bioinformaticians developing new tools using our resourceful libraries.

1. **Training on mass spectrometry data.** CIBI offers various courses to introduce mass spectrometry data analysis with a focus on proteomics and metabolomics. In these workshops, concepts such as non-targeted label-free analysis are taught and users learn how to implement complex analysis workflows based on OpenMS tools with subsequent visualisation of the results based on real-life data. In addition to quantification analysis, metabolite identification with MetFrag ^34^ and MetFamily are taught on several events. The target audience are mainly beginners and intermediate users of proteomics and metabolomics that want to learn how to efficiently process their mass spectrometry data.
2. **Training on sequencing data.** Another major field in which CIBI offers various training activities relates to sequencing data analysis. Training courses are focused on two user groups. The first are bioinformaticians developing new tools. In dedicated hands-on sessions on the annual user meeting, bioinformaticians are taught how to use the SeqAn software library to write new competitive tools using state-of-the-art algorithms and data structures. The offered trainings mostly focus on beginners but require an intermediate degree of programming knowledge. Knowledge in C++ is helpful but not required. For advanced SeqAn users, we host an annual developer meeting where we tackle specific problems of the participants and add new components to the library. The second target group are data scientists. In these courses, data scientists explore typical analysis pipelines using SeqAn and external tools integrated into the KNIME analytics platform.
3. **Training on bioimage data.** Annual hands-on courses introduce participants to develop within and for the Fiji ^35^, ImageJ2 ^36, 37^, and KNIME ^38^ ecosystems. In addition, CIBI offers a deep learning course especially for image-based problems.
4. **Training on workflows and tool integration.** CIBI organizes life science workshops on the KNIME Spring Summit and teaches the integration of our tools in KNIME to produce strong and efficient multi-omics pipelines on various meetings (either on international conferences or on joint user meetings). Hence, many of the CIBI events are accompanied with experts in KNIME and users are always welcome to bring their own data so CIBI experts can develop an efficient solution for their problem at hand.
5. **Training on conferences.** CIBI offers one-day workshops on international conferences, where the CIBI members can get in touch with life scientists around the world and discuss the topical research subjects and how to tackle them with our software portfolios.
6. **Online training.** CIBI made most of the training materials available online as self-paced training. These include written tutorials and videos with many practical tips and examples. The materials are intended for self-regulated learning to study and explore the capabilities of our software and tools in a comfortable way. The target audience of these materials ranges from novices to advanced users.
7. **Hackathons and developer meetings.** For all projects and topics, CIBI offers annual hackathons and developer meetings. During these meetings, specific problems and future directions of our software are discussed and implementation of feature requests are tackled together with the community.

### RNA Bioinformatics Center (RBC)

The RNA Bioinformatics Center (RBC) brings together all major RNA bioinformatics groups in Germany located at three sites: University of Freiburg, University of Leipzig and Max Delbrück Center in Berlin. Since November 2016, RBC includes two partner projects called de.STAIR (Structured Analysis and Integration of RNA-Seq experiments) and de.NBI-epi (Computational Epigenetics).

***Research focus.*** The RNA Bioinformatics Center (RBC) deals with all RNA-related data not limited to transcriptome analysis but also RNA structure analysis, prediction of targets of RBPs (CLIP-Seq) and non-coding RNAs, definition and classification of RNA transcripts and the analysis of protein-RNA and RNA-RNA interactions. The RBC-Freiburg focuses on the analysis of RNA-RNA and RNA-protein interactions ^39^, RBC-Leipzig on the analysis of non-coding RNAs and RNA structure ^40^ and RBC-Berlin on RNA-binding proteins and post-transcriptional regulation ^41^. The partner project de.STAIR consists of three sites: de.STAIR-Freiburg working on the regulatory RNA interaction and integration, de.STAIR-Jena on the causes and effects of quantitative and qualitative expression changes and de.STAIR-Rostock on RNA-Seq workflow specification and technical integration ^42^. The second partner project de.NBI-epi is located in Berlin and in Freiburg and is focusing on Computational Epigenetics. 

The center maintains and develops the largest Galaxy instance in Europe with more than 12000 users, responsible for more than 10 million submitted jobs. The Galaxy server is freely open to all researchers via useGalaxy.eu. RBC maintains an integrated, easily accessible RNA analysis workbench ^43^, based on Galaxy, which can be downloaded and installed locally, or deployed on HPC-like environments or clouds. It includes more than 50 RNA tools, multiple workflows, interactive tours and training data.

***Training and outreach activities.*** With its wider focus on the analysis of high-throughput data in Galaxy, an important goal of the RBC is to support researchers by educating them in big data analysis, programming, data management, Galaxy server administration and more. The RBC members believe that sharing of knowledge and the open science movement are the key points for the future success in the analysis of life science data. For that reason, the RBC and its partner projects provide training events covering diverse topics (data analysis, programming, tool development, containers, etc.) and targeting a diverse audience at different levels of experience (scientists, developers, administrators, trainers - from beginners to experts).

1. **Training on HTS and RNA-related data.** For example, RBC-Freiburg is offering a full-week hands-on HTS data analysis workshop in Freiburg twice per year: Introduction to Galaxy and HTS, RNA-Seq, ChIP-Seq, Exome-Seq, MethylC-Seq, etc. Similar courses are also available at the RBC-Berlin site. Many other training workshops are also given in collaboration with other de.NBI centers and ELIXIR, not only in Germany and Europe but also around the world.
2. **Galaxy Training Network (GTN).** As face-to-face workshops do not fit to the scale of demand, the RBC has put a lot of effort into developing and offering online training material. RBC-Freiburg is a major contributor to the community-driven development of Galaxy training material ^2^. All of this material is freely accessible under a Creative Commons license at https://training.galaxyproject.org. It contains tutorials with hands-on, slides and interactive tours, designed for both self-training and workshops, as well as the technical support with tools, data, virtualized instances, etc. RBC-Freiburg via useGalaxy.eu is also offering a special service for Galaxy trainers: Training Infrastructure as a Service (TIaaS: https://galaxyproject.eu/tiaas), completely dedicated compute resources for the duration of a workshop training.
3. **Mentorship.** Mentorship is also a practice in place at RBC. For example, RBC-Freiburg has guests regularly, who would like to learn a technology or gain specific knowledge by immersion for a few days. The mentorship also works remote via support on real-time chat and also via online meeting with other instructors (as in the Carpentries) or developers. Mentorship programs can be requested via an online booking system. RBC, in collaboration with HD-HuB and ELIXIR-UK, is also building a mentoring program for life-scientists on open science as part of the Mozilla Open Leader program.
4. **Hackathons.** To combine forces, RBC regularly organizes hackathons and contribution fests: short events (usually few days) where people work together to develop new, or improving existing techniques, tools, training materials, etc. Several have been organized per year on site or online, in close cooperation with de.NBI, ELIXIR, and worldwide communities, like Bioconda.
5. **Administrator training.** RBC is offering multiple workshops dedicated to administrators during a year. That involves administering servers, creating and setting up Virtual Research Environments (VRE), deploying VREs, managing cloud deployments, containers and monitoring. In a weeklong workshop, administrators can also learn how to deploy production ready Galaxy servers that scale to multiple thousands of users.
6. **Local outreach activities.** RBC is also involved in an outreach program, Street Science Community, supported by de.NBI. The aim of this program is to bring science, in particular life science, to schools and to the general public via workshops during which participants can extract and sequence DNA of yeast from beers.

### German Crop BioGreenformatics Network (GCBN)

The German CropBioGreenformatics Network (GCBN) is a collaboration between three partners: Leibniz Institute of Plant Genetics and Crop Plant Research (IPK) Gatersleben, German Research Center for Environmental Health Munich and Forschungszentrum Jülich.

***Research focus.*** GCBN provides crop plant-related bioinformatics services in the field of green bioinformatics. GCBN provides transparent access to germplasm data, it provides tools to annotate plant genes, genomes and transposons and is collaborating with plant phenotyping centers (e.g. the ESFRI structure EMPHASIS) ^44^. GCBN uses this interaction to help in bridging genotypes with phenotypes, i.e. to predict plant phenotypes from genotypes or to analyze plants, which are preserved in genebanks ^45^. As such, GCBN is also involved in plant phenotype standardization together with the EMBL-EBI and all GCBN partners are involved in the ELIXIR plant community activities.

***Training and outreach activities.*** Together with other de.NBI partners, GCBN organizes different training courses. GCBN consults and provides help to users from plant sciences and breeding companies. Given the expertise within GCBN, the main focus is on large scale data processing in the plant sciences, focusing on plant omics data such as metabolomics and transcriptomics data, genome assemblies and annotation, plant phenotyping data and the interaction between the plant genome(s) and the resulting phenotype.

1. **Computational biology starter.** GCBN offers introductory courses into computational plant biology. These courses usually introduce the basic use of Linux and R as well specific tools for plant phenotyping and data analysis.
2. **Training on RNA-Seq for plants.** RNA-Seq courses for plants focus on the quantitative analysis of plant RNA-Seq data from read mapping to data visualization using e.g. MapMan ^46, 47^. The courses focus on the specific issues of plant transcriptomics data such as polyploid genome and ancient gene duplications and their effect on expression data.
3. **Training on phenotyping analysis.** Plant phenotyping data and analysis courses are usually run in conjunction with the German Plant Phenotyping Network DPPN or the EMPHASIS pan-European Infrastructure. Here, scientists learn about data standards, extraction of data from images and data normalization.
4. **Training on FAIR Data.** GCBN contributes to FAIR (Findable, Accessible, Interoperable, Reusable) data workshops held by other service centers and mainly by the BioData service center. Furthermore, it is contributing the plant specific topics and FAIRification in the plant sciences especially for data provision in repositories like e!DAL-PGP ^48^.
5. **Training on handling Nanopore data.** GCBN contributes to the Nanopore best practice workshops of BiGi (see earlier subsection on BiGi). Here, GCBN provides its expertise on long read data generation, cleaning and assembly for complex, repetitive plant genomes.

### Center for Biological Data (BioData)

The BioData service center is a collaboration of five partners: Jacobs University Bremen, Alfred-Wegener-Institut - Helmholtz Zentrum für Polar- und Meeresforschung, MARUM/University of Bremen, Leibniz Institute DSMZ, Technical University of Braunschweig and the partner project at the University of Hamburg.

***Research focus.*** The BioData service center consists of the information systems SILVA ^49^, PANGAEA ^50^, BacDive ^51^, BRENDA ^52^ and ProteinsPlus. BioData provides highly curated reference datasets and related services for users in academia and industry. The BioData service center facilitates access to services for ribosomal RNA genes (SILVA), georeferenced data from earth system and environmental research (PANGAEA), detailed strain-linked information on the different aspects of bacterial and archaeal biodiversity (BacDive), comprehensive information on all aspects of enzyme functions (BRENDA), as well as easily accessible protein structure data (ProteinsPlus). SILVA and BRENDA have been identified as ELIXIR Core Data Resources underlining their fundamental importance to the wider life-science community and the long-term preservation of biological data in Europe and beyond ^53, 54^.

***Training and outreach activities.*** The BioData service center concentrates on data products as well as on research and services. It is well known for its reference databases for taxonomy, phylogeny, biotechnology, biochemistry, pharmacy, medicine, quality control, diagnostics, environmental and biodiversity research. BioData provides analysis services for enzyme structures and functions, classification of next-generation sequencing data. In addition, BioData is highly active in data mobilization and publishing as well as research data management following the FAIR ^55^ (Findable, Accessible, Interoperable, Reusable) data principles. Therefore, the targeted audience are researchers in the life- or medical sciences, who like to use data and data products as references in their research or applications and/or manage and publish their data in the spirit of science 2.0 aka open-science.

1. **Training on FAIR data.** The BioData training courses include the topic ‘FAIR data’, especially within the de.NBI summer school 2018. Here, the courses raise awareness for the importance of proper research data management in general, but also provide a practical toolbox for the acquisition, curation, documentation, archiving and publication of research data following the FAIR data principles. Furthermore, practical training on accessing the data for re-use and integration are part of their training courses.
2. **Training on databases.** The BioData service center bundles large databases and provides the corresponding training courses for these databases. For instance, in the training course on the rDNA reference database SILVA, in addition, all necessary steps researchers need to take when performing amplicon-based investigations using the rDNA as a marker gene are explained. The course starts with experimental design, including an overview of relevant sequencing technologies, and the selection, design, and evaluation of primers for the amplification of rDNA. It provides examples and best practice solutions for data pre-processing and quality assurance up to the contextualized submission of the NGS data to public repositories. It provides a framework for statistical analysis of the data including a short Linux and R crash course. In addition, this course also combines an introduction to the BacDive database (Bacterial Diversity Metadatabase) as a tool for further data integration to better understand the biology of sequences/organisms analyzed.

### de.NBI Systems Biology Service center (de.NBI-SysBio)

de.NBI Systems Biology service center (de.NBI-SysBio) is the service center supporting particularly the systems biology community by providing data and model management tools, manually curated reaction kinetics data and world-wide recognized tools for mathematical modeling of biological networks. The data management part of de.NBI-SysBio is provided by the HITS (Heidelberg Institute for Theoretical Studies). The partner project de.NBI-ModSim ( Heidelberg University and the Max Planck Institute for Dynamics of Complex Technical Systems Magdeburg) deliver support in model construction.

***Research focus.*** The data management part of de.NBI-SysBio has the mission to serve customers with standardized high-quality scientific data and data services. The data management system “SEEK for Science” has been developed by the transnational FAIRDOM project ^5^ as a sharing space for projects fitted to the needs of systems and synthetic biology as well as systems medicine. The database SABIO-RK provides structured, standardized and annotated kinetic data with a focus on supporting the computational modelling community to create models of biochemical reaction networks as well as on allowing experimentalists to gain further knowledge about enzymatic activities and reaction properties. The data coming mainly from literature are expert-curated and can be easily exported for direct use, e.g. in modeling tools like COPASI ^54^ to run simulations.

The modeling part of de.NBI-SysBio is provided by Heidelberg University and the Max Planck Institute for Dynamics of Complex Technical Systems Magdeburg and their tools COPASI ^56^ and CellNetAnalyzer ^57^. CellNetAnalyzer (CNA) is a MATLAB toolbox serving a graphical user interface and various computational methods and algorithms for exploring structural and functional properties of metabolic, signaling, and regulatory networks. COPASI is a widely used software tool for creating, simulating and analysing models of biochemical reaction networks.

***Training and outreach activities.*** The de.NBI-SysBio training courses, workshops and tutorials are suitable for beginners as well as for advanced users. For instance, the three-days training course ‘Tools for systems biology modeling and data exchange: COPASI, CellNetAnalyzer, SABIO-RK, SEEK’ with extensive hands-on sessions annually takes place alternately in Heidelberg or Magdeburg. Additionally, online tutorials are offered to introduce the main tools (e.g. FAIRDOMHub ^5^, SABIO-RK ^58^, COPASI ^56^, CellNetAnalyzer ^57^).

As regular international outreach activities de.NBI-SysBio is involved in the organization of satellites of the annual ICSB conference (International Conference on Systems Biology) with the ‘COMBINE Tutorial - Modelling and Simulation Tools in Systems Biology” and “Advanced Modeling with COPASI’.

1. **Training on data management and FAIR data.** de.NBI-SysBio was involved in organization of the de.NBI summer school on FAIR data and data management in 2018. The data management training courses are open to everyone interested in producing FAIR data in project driven scientific research. de.NBI-SysBio offers special training courses for experimentalists or for modelers but also more general workshops and tutorials with breakout sessions to delve into the topic from a modeler’s, experimentalist’s or developer’s point of view (e.g. annual Systems Biology Developers Foundry).
  Furthermore, de.NBI-SysBio offers on demand visits to support customers in installing their projects or local SEEK instance.
2. **Training on modeling.** The trainings for the modeling tools CellNetAnalyzer and COPASI offered by de.NBI-ModSim aim at modelers or those who want to become one. Attendees learn basic model construction and analysis techniques for kinetic modeling of biochemical systems (illustrated and exercised with COPASI) and principles of stoichiometric and constraint-based modeling of metabolic networks (coupled with hands-on exercises using CellNetAnalyzer).

## de.NBI Cloud-based training

In order to perform analysis on datasets available in the life sciences, an appropriate compute infrastructure is necessary. The de.NBI Cloud was created to fill this gap and offers storage and compute resources for researchers in life science in Germany. Through a cloud federation setup, the six cloud sites including Bielefeld, Gießen, Freiburg, Heidelberg, Berlin and Tübingen are integrated into a single cloud platform and offer more than 33000 cores and 38 PB of storage capacity in total. A single sign-on (SSO) mechanism enables the user to access any de.NBI Cloud service *via* their home institution account using the ELIXIR authentication and authorization infrastructure system (ELIXIR AAI) and in particular Perun, the identity and access management system of ELIXIR ^17^.

### Technical aspects

Currently, the de.NBI Cloud offers the project types SimpleVM and OpenStack ^17^. Both project types are using virtual machines (VM), where a VM is an emulation of an operating system and represents one of the main building blocks of the cloud. OpenStack is an *Infrastructure as a Service* (IAAS) system that allows a developer to configure any computational resources like networking, storage and VM settings for running large-scale analyses or offering of a web service in the cloud for other researchers. SimpleVM is a beginner friendly project type that streamlines the handling of VMs and does not demand any particular knowledge in Cloud Computing. While OpenStack is suitable for training in the context of Big Data like running workflows for metagenome analysis, the SimpleVM project type can be used for command line, Linux or any other trainings, where running tools on one virtual machine is sufficient.

### Application process and getting started

The procedure for applying, using and accessing computational resources for a de.NBI Cloud training course is the following. First, a principal investigator (PI) of a German university or research institution must apply for a project through the de.NBI Cloud portal. As soon as the access committee approves the application, the PI can add the actual trainer and set him as an administrator of the project. The trainer first starts a VM by choosing an appropriate Linux system, e.g. Ubuntu or CentOS, and then installs any software needed by the course participants. Once the trainer has installed the required software, the VM can be snapshotted and made accessible to all course participants. At the beginning of the training course, the PI adds further course participants, who can use exactly the same tools based on the initial VM created by the trainer. Finally, the trainer and the participants can directly start with the actual course.

### Training activities

Since the beginning of 2018, the de.NBI Cloud is in production and multiple workshops, tutorials and even a user meeting have been organized. The given workshops range from research related topics like Nanopore workshops to more cloud computing related topics.

1. **Training on sequencing data.** In Nanopore best practice workshops participants are taught about Nanopore sequencing technology, its applications and the “Best Practice” bioinformatics workflow (see subsections on BiGi and GCBN). Nanopore tools such as long read assemblers demand many cores and especially a lot of RAM that can be made available by the de.NBI Cloud.
2. **Training on metagenomics tools.** Other training events like metagenome workshops are demonstrating how to store and analyze large datasets using state of the art bioinformatic tools in the cloud (see subsection on BiGi). These workshops highlight one of the main use cases of the cloud, which is the handling of Big Data.
3. **Summer schools and user meetings.** Cloud Computing related tutorials and lectures are given during Summer Schools and the de.NBI Cloud User Meeting. In September 2018, de.NBI Cloud organized the first de.NBI Cloud User Meeting in order to teach users best practices in Cloud Computing and to allow them to meet Cloud Computing experts. The user meeting offered presentations about use cases and hands-on sessions for learning new technologies and best practices in handling data, tools and workflows. Workshops ranged from introductions into OpenStack but also covered service-oriented areas like e.g. Kubernetes. Due to the positive feedback received from the attendees, the second user meeting took place in Heidelberg in September 2019.

### The role of de.NBI / ELIXIR-DE in the ELIXIR training platform

In 2016, Germany officially joined ELIXIR, the European life sciences infrastructure for biological information. The ELIXIR-DE node is operated by de.NBI and consequently, de.NBI and SIG 3 joined the ELIXIR training platform. SIG 3 already started to establish collaborations with ELIXIR in training activities and discussion on the exchange of data for training events on both the technical and administrative level, e.g. ELIXIR-DE co-organized the European Galaxy Developer Workshop 2017 in collaboration with other ELIXIR nodes. All de.NBI training course belong to the ELIXIR training program and are available on ELIXIR’s Training Portal, TeSS. Therefore, the user feedback of these courses in form of a standardized survey is collected by ELIXIR and included into the general ELIXIR training feedback ^59^. In addition, SIG 3 and the ELIXIR Germany training coordinators (chair and deputies) are part of a new e-learning working group and of the FAIR training working group ( https://github.com/elixir-europe/FAIR-Training). They participate in two implementation studies (Software/Data Carpentries & Cloud for Training), the annual ELIXIR BioHackathon and in different training activities of ELIXIR Communities. With the ELIXIR Staff Exchange Program for Galaxy Train-the-Trainer (TtT) events, SIG 3 can exchange knowledge and training skills with the different ELIXIR training communities and aim to build and provide high quality training resources. In association with members from the ELIXIR training network, the Galaxy Training Network and The Carpentries, SIG 3 also contribute to building a full curriculum on the computational analysis of high throughput sequencing data and runs the two corresponding hybrid workshops. As part of the Mozilla Open Leader program and in collaboration with the ELIXIR Training Platform, SIG 3 is also building a mentoring program on open science for early life-scientists in Europe. With the involvement in the e-learning working group, the e-learning support for bioinformatics in Germany will be improved by integrating different methods and other implementations studies within ELIXIR.

## Future plans and conclusion

To show the success of the de.NBI training program for Germany, the progress in different types of training / education activities as well as the performance has to be summarized. First, the de.NBI summer schools provided training courses (primarily for undergraduate and graduate students) in specific topics related to one or several de.NBI service centers. The de.NBI summer schools on Microbial Bioinformatics organized by BiGi, RBC and de.NBI-SysBio in September 2015, on Proteomics by BioInfra.Prot, CIBI and BiGi in September 2016 and on Computational genomics and RNA biology by RBC in September 2017 were very successful in terms of applications (applicants 2015: 36; 2016: 53; 2017: 60). In addition, de.NBI also organized a summer school on cloud computing in Gießen in June 2017 and a winter school on metabolomics in March 2018. A summer school on FAIR data and data management ‘Riding the Data Life Cycle’ was organized in September 2018 (applicants 2018: 27), whereas in 2019, a summer school on (Bio)Data Science took place in September (applicants in 2019: 30) in Gatersleben. Due to the COVID-19 pandemic in Germany, summer school 2020 was postponed to spring 2021. For 2021, the spring school topic will be Metagenomics, whereas the summer school will focus on Proteomics & Lipidomics.

Tool- and topic-specific training was also extended in the last few years ( Table 1). In 2015, 17 training courses with 329 participants were organized by de.NBI. In 2016, the network organized 40 training events with 882 participants. The new established training courses closed gaps in the topics covered in 2015 and were adapted to cover a broader range of qualification levels (from beginner to expert). In 2017, de.NBI further raised in the number of courses and participants (69 training courses with 1489 participants). In 2018, the number of training events plateau at 1520 participants and 77 courses. In 2019, de.NBI further increased the number of courses and participants (79 training courses with 1,586 participants). For the year 2020, a similar number of courses was planned to keep the high-quality standards of de.NBI training courses. Based on new external partners in the training field, a slight increase in training courses and participants was expected. Due to the COVID-19 pandemic in Germany, all training courses between March and May were canceled.

**Table 1.** de.NBI training course statistics 2015–2019 including courses, applicants and participants.

	Training courses	Applicants	Participants
2015	17	551	329
2016	40	1177	882
2017	69	1679	1489
2018	77	1853	1520
2019	79	2258	1586

**Figure 2.**
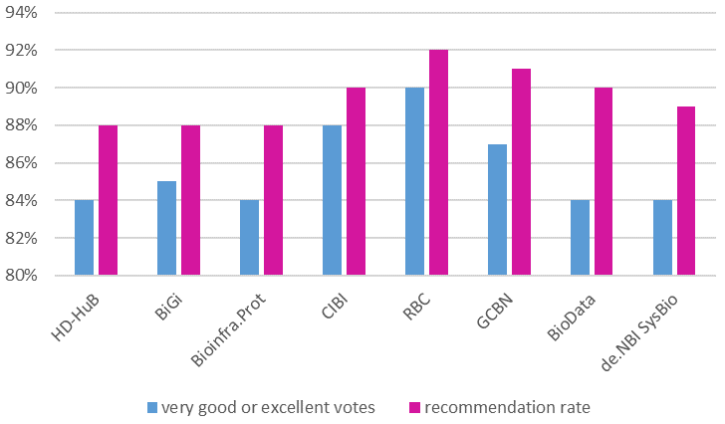
Performance of de.NBI training courses 2015–2019 regarding user satisfaction and recommendations in each service center.

Regarding performance, de.NBI and ELIXIR-DE guarantee high quality standards (~90% recommendation rate, ~86% very good or excellent votes based on about 2000 feedback responses - July 2020) above ELIXIR average (~89% recommendation rate, ~70% very good or excellent votes based on about 8500 feedback responses – July 2020) ( Figure 2). However, a general comparison of the training programs provided by each ELIXIR node is very difficult. Every country has a specific teaching focus and different amounts of training courses. In general, ELIXIR UK and ELIXIR Switzerland offer a broad portfolio of training courses similar to de.NBI / ELIXIR-DE ^60, 61^, whereas smaller ELIXIR nodes, like Estonia, are only able to offer a few courses including the main research aspects in their countries.

In addition to face-to-face training, online training was introduced on the de.NBI website in 2016. Further, online hackathons for different software packages were established by the service center RBC in 2016. In 2019, the repertoire of online material was increased to 45 items and guidelines for online training were developed by SIG 3. In 2020, further online material will be provided by the different service centers to reach about 60 items - and thus a larger audience that cannot be covered by the training courses alone.

To sum up the effort of the de.NBI / ELIXIR-DE training platform: Since 2015, de.NBI service centers, the de.NBI Administration Office and SIG 3 worked very successfully together to establish, coordinate and expand bioinformatics training courses across Germany and in ELIXIR. In total, about 6,000 participants (Status July 2020) were trained in different bioinformatics disciplines to tackle the challenges of the five Vs of the big data problem.

Nevertheless, training still has a high priority for de.NBI / ELIXIR-DE and significant future efforts will be put into the creation of more e-learning material, the recruitment of new external de.NBI training partners and the qualification of trainers to keep pushing training activities forward and to continue participating in the ELIXIR training platform.

## Data availability

No data are associated with this article.

## References

[1] Higdon R Haynes W Stanberry L: Unraveling the Complexities of Life Sciences Data. *Big Data.* 2013;1(1):42–50. 10.1089/big.2012.1505 27447037

[2] Batut B Hiltemann S Bagnacani A: Community-Driven Data Analysis Training for Biology. *Cell Syst.* 2018;6(6):752–758. 10.1016/j.cels.2018.05.012 29953864 PMC6296361

[3] Tauch A Al-DIlaimi A: Bioinformatics in Germany: toward a national-level infrastructure. *Brief Bioinform.* 2019;20(2):370–374. 10.1093/bib/bbx040 28430873 PMC6433733

[4] Stevens SLR Kuzak M Martinez C: Building a local community of practice in scientific programming for life scientists. *PLoS Biol.* 2018;16(11):e2005561. 10.1371/journal.pbio.2005561 30485260 PMC6287879

[5] Wittig U Rey M Weidemann A: Data management and data enrichment for systems biology projects. *J Biotechnol.* 2017;261:229–237. 10.1016/j.jbiotec.2017.06.007 28606610

[6] Reisinger E Genthner L Kerssemakers J: OTP: An automatized system for managing and processing NGS data. *J Biotechnol.* 2017;261:53–62. 10.1016/j.jbiotec.2017.08.006 28803971

[7] Schmidt TSB Raes J Bork P: The Human Gut Microbiome: From Association to Modulation. *Cell.* 2018;172(6):1198–1215. 10.1016/j.cell.2018.02.044 29522742

[8] Rauscher B Valentini E Hardeland U: Phenotype databases for genetic screens in human cells. *J Biotechnol.* 2017;261:63–69. 10.1016/j.jbiotec.2017.06.008 28625679

[9] Wollmann T Erfle H Eils R: Workflows for microscopy image analysis and cellular phenotyping. *J Biotechnol.* 2017;261:70–75. 10.1016/j.jbiotec.2017.07.019 28757289

[10] Durek P Nordström K Gasparoni G: Epigenomic Profiling of Human CD4 ^+^ T Cells Supports a Linear Differentiation Model and Highlights Molecular Regulators of Memory Development. *Immunity.* 2016;45(5):1148–1161. 10.1016/j.immuni.2016.10.022 27851915

[11] Meyer F Goesmann A McHardy AC: GenDB--an open source genome annotation system for prokaryote genomes. *Nucleic Acids Res.* 2003;31(8):2187–95. 10.1093/nar/gkg312 12682369 PMC153740

[12] Blom J Albaum SP Doppmeier D: EDGAR: A software framework for the comparative analysis of prokaryotic genomes. *BMC Bioinformatics.* 2009;10:154. 10.1186/1471-2105-10-154 19457249 PMC2696450

[13] Blom J Kreis J Spänig S: EDGAR 2.0: an enhanced software platform for comparative gene content analyses. *Nucleic Acids Res.* 2016;44(w1):w22–8. 10.1093/nar/gkw255 27098043 PMC4987874

[14] Yu J Blom J Glaeser SP: A review of bioinformatics platforms for comparative genomics. Recent developments of the EDGAR 2.0 platform and its utility for taxonomic and phylogenetic studies. *J Biotechnol.* 2017;261:2–9. 10.1016/j.jbiotec.2017.07.010 28705636

[15] Jünemann S Kleinbölting N Jaenicke S: Bioinformatics for NGS-based metagenomics and the application to biogas research. *J Biotechnol.* 2017;261:10–23. 10.1016/j.jbiotec.2017.08.012 28823476

[16] Heyer R Schallert K Zoun R: Challenges and perspectives of metaproteomic data analysis. *J Biotechnol.* 2017;261:24–36. 10.1016/j.jbiotec.2017.06.1201 28663049

[17] Belmann P Fischer B Krüger J: de.NBI Cloud federation through ELIXIR AAI. *F1000Research.* 2019;8:842. 10.12688/f1000research.19013.1 31354949 PMC6635982

[18] Linden M Prochazka M Lappalainen I: Common ELIXIR Service for Researcher Authentication and Authorisation. *F1000Research.* 2018;7. 10.12688/f1000research.15161.1 30254736 PMC6124379

[19] Schwengers O Hoek A Fritzenwanker M: ASA ^3^P: An automatic and scalable pipeline for the assembly, annotation and higher level analysis of closely related bacterial isolates. *bioRxiv.* 2019 10.1101/654319 PMC7077848 32134915

[20] Brink BG Seidel A Kleinbölting N: Omics Fusion - A Platform for Integrative Analysis of Omics Data. *J Integr Bioinform.* 2016;13(4):296. 10.2390/biecoll-jib-2016-296 28187412

[21] Muth T Behne A Heyer R: The MetaProteomeAnalyzer: A powerful open-source software suite for metaproteomics data analysis and interpretation. *J Proteome Res.* 2015;14(3):1557–65. 10.1021/pr501246w 25660940

[22] Turewicz M Kohl M Ahrens M: BioInfra.Prot: A comprehensive proteomics workflow including data standardization, protein inference, expression analysis and data publication. *J Biotechnol.* 2017;261:116–125. 10.1016/j.jbiotec.2017.06.005 28606611

[23] Uszkoreit J Maerkens A Perez-Riverol Y: PIA: An Intuitive Protein Inference Engine with a Web-Based User Interface. *J Proteome Res.* 2015;14(7):2988–97. 10.1021/acs.jproteome.5b00121 25938255

[24] Turewicz M Ahrens M May C: PAA: an R/bioconductor package for biomarker discovery with protein microarrays. *Bioinformatics.* 2016;32(10):1577–9. 10.1093/bioinformatics/btw037 26803161 PMC4866526

[25] Barsnes H Vaudel M: SearchGUI: A Highly Adaptable Common Interface for Proteomics Search and de Novo Engines. *J Proteome Res.* 2018;17(7):2552–2555. 10.1021/acs.jproteome.8b00175 29774740

[26] Kopczynski D Sickmann A Ahrends R: Computational proteomics tools for identification and quality control. *J Biotechnol.* 2017;261:126–130. 10.1016/j.jbiotec.2017.06.1199 28676234

[27] Vaudel M Burkhart JM Zahedi RP: PeptideShaker enables reanalysis of MS-derived proteomics data sets. *Nat Biotechnol.* 2015;33(1):22–4. 10.1038/nbt.3109 25574629

[28] Schwudke D Shevchenko A Hoffmann N: Lipidomics informatics for life-science. *J Biotechnol.* 2017;261:131–136. 10.1016/j.jbiotec.2017.08.010 28822794

[29] Herzog R Schwudke D Shevchenko A: LipidXplorer: Software for Quantitative Shotgun Lipidomics Compatible with Multiple Mass Spectrometry Platforms. *Curr Protoc Bioinforma.* 2013;43(1):14.12.1-30. 10.1002/0471250953.bi1412s43 26270171

[30] Marella C Torda AE Schwudke D: The LUX Score: A Metric for Lipidome Homology. *PLoS Comput Biol.* 2015;11(9):e1004511. 10.1371/journal.pcbi.1004511 26393792 PMC4578897

[31] Pfeuffer J Sachsenberg T Alka O: OpenMS - A platform for reproducible analysis of mass spectrometry data. *J Biotechnol.* 2017;261:142–148. 10.1016/j.jbiotec.2017.05.016 28559010

[32] Reinert K Dadi TH Ehrhardt M: The SeqAn C++ template library for efficient sequence analysis: A resource for programmers. *J Biotechnol.* 2017;261:157–168. 10.1016/j.jbiotec.2017.07.017 28888961

[33] Fillbrunn A Dietz C Pfeuffer J: KNIME for reproducible cross-domain analysis of life science data. *J Biotechnol.* 2017;261:149–156. 10.1016/j.jbiotec.2017.07.028 28757290

[34] Meier R Ruttkies C Treutler H: Bioinformatics can boost metabolomics research. *J Biotechnol.* 2017;261:137–141. 10.1016/j.jbiotec.2017.05.018 28554829

[35] Schindelin J Arganda-Carreras I Frise E: Fiji: an open-source platform for biological-image analysis. *Nat Methods.* 2012;9(7):676–82. 10.1038/nmeth.2019 22743772 PMC3855844

[36] Pietzsch T Preibisch S Tomancák P: ImgLib2--generic image processing in Java. *Bioinformatics.* 2012;28(22):3009–11. 10.1093/bioinformatics/bts543 22962343 PMC3496339

[37] Rueden CT Schindelin J Hiner MC: ImageJ2: ImageJ for the next generation of scientific image data. *BMC Bioinformatics.* 2017;18(1):529. 10.1186/s12859-017-1934-z 29187165 PMC5708080

[38] Dietz C Berthold MR: KNIME for Open-Source Bioimage Analysis: A Tutorial. *Adv Anat Embryol Cell Biol.* 2016;219:179–97. 10.1007/978-3-319-28549-8_7 27207367

[39] Backofen R Engelhardt J Erxleben A: RNA-bioinformatics: Tools, services and databases for the analysis of RNA-based regulation. *J Biotechnol.* 2017;261:76–84. 10.1016/j.jbiotec.2017.05.019 28554830

[40] Fallmann J Will S Engelhardt J: Recent advances in RNA folding. *J Biotechnol.* 2017;261:97–104. 10.1016/j.jbiotec.2017.07.007 28690134

[41] Wreczycka K Gosdschan A Yusuf D: Strategies for analyzing bisulfite sequencing data. *J Biotechnol.* 2017;261:105–115. 10.1016/j.jbiotec.2017.08.007 28822795

[42] Lott SC Wolfien M Riege K: Customized workflow development and data modularization concepts for RNA-Sequencing and metatranscriptome experiments. *J Biotechnol.* 2017;261:85–96. 10.1016/j.jbiotec.2017.06.1203 28676233

[43] Grüning BA Fallmann J Yusuf D: The RNA workbench: best practices for RNA and high-throughput sequencing bioinformatics in Galaxy. *Nucleic Acids Res.* 2017;45(W1):W560–W566. 10.1093/nar/gkx409 28582575 PMC5570170

[44] Schmutzer T Bolger ME Rudd S: Bioinformatics in the plant genomic and phenomic domain: The German contribution to resources, services and perspectives. *J Biotechnol.* 2017;261:37–45. 10.1016/j.jbiotec.2017.07.006 28698099

[45] Mascher M Schreiber M Scholz U: Genebank genomics bridges the gap between the conservation of crop diversity and plant breeding. *Nat Genet.* 2019;51(7):1076–1081. 10.1038/s41588-019-0443-6 31253974

[46] Schwacke R Ponce-Soto GY Krause K: MapMan4: A Refined Protein Classification and Annotation Framework Applicable to Multi-Omics Data Analysis. *Mol Plant.* 2019;12(6):879–892. 10.1016/j.molp.2019.01.003 30639314

[47] Usadel B Poree F Nagel A: A guide to using MapMan to visualize and compare Omics data in plants: a case study in the crop species, Maize. *Plant Cell Environ.* 2009;32(9):1211–29. 10.1111/j.1365-3040.2009.01978.x 19389052

[48] Arend D Junker A Scholz U: PGP repository: a plant phenomics and genomics data publication infrastructure. *Database (Oxford).* 2016;2016:baw033. 10.1093/database/baw033 27087305 PMC4834206

[49] Glöckner FO Yilmaz P Quast C: 25 years of serving the community with ribosomal RNA gene reference databases and tools. *J Biotechnol.* 2017;261:169–176. 10.1016/j.jbiotec.2017.06.1198 28648396

[50] Diepenbroek M Schindler U Huber R: Terminology supported archiving and publication of environmental science data in PANGAEA. *J Biotechnol.* 2017;261:177–186. 10.1016/j.jbiotec.2017.07.016 28743591

[51] Reimer LC Söhngen C Vetcininova A: Mobilization and integration of bacterial phenotypic data-Enabling next generation biodiversity analysis through the BacDive metadatabase. *J Biotechnol.* 2017;261:187–193. 10.1016/j.jbiotec.2017.05.004 28487186

[52] Schomburg I Jeske L Ulbrich M: The BRENDA enzyme information system-From a database to an expert system. *J Biotechnol.* 2017;261:194–206. 10.1016/j.jbiotec.2017.04.020 28438579

[53] Drysdale R Cook CE Petryszak R: The ELIXIR Core Data Resources: fundamental infrastructure for the life sciences. *bioRxiv.* 2019 10.1101/598318 PMC7446027 31950984

[54] Jeske L Placzek S Schomburg I: BRENDA in 2019: a European ELIXIR core data resource. *Nucleic Acids Res.* 2019;47(D1):D542–D549. 10.1093/nar/gky1048 30395242 PMC6323942

[55] Harjes J Triebel D Link A: FAIR data in meta-omics research: Using the MOD-CO schema to describe structural and operational elements of workflows from field to publication. *Biodivers Inf Sci Stand.* 2019;e37596 10.3897/biss.3.37596

[56] Bergmann FT Hoops S Klahn B: COPASI and its applications in biotechnology. *J Biotechnol.* 2017;261:215–220. 10.1016/j.jbiotec.2017.06.1200 28655634 PMC5623632

[57] von Kamp A Thiele S Hädicke O: Use of CellNetAnalyzer in biotechnology and metabolic engineering. *J Biotechnol.* 2017;261:221–228. 10.1016/j.jbiotec.2017.05.001 28499817

[58] Krebs O Golebiewski M Kania R: SABIO-RK: A data warehouse for biochemical reactions and their kinetics. *J Integr Bioinform.* 2007;4(1). 10.1515/jib-2007-49

[59] Gurwitz KT Gaur PS Bellis LJ: A framework to assess the quality and impact of bioinformatics training across ELIXIR. *PLoS Comput Biol.* 2020;16(7):e1007976. 10.1371/journal.pcbi.1007976 32702016 PMC7377377

[60] Larcombe L Hendricusdottir R Attwood TK: ELIXIR-UK role in bioinformatics training at the national level and across ELIXIR. *F1000Res.* 2017;6:ELIXIR-952. 10.12688/f1000research.11837.1 28781748 PMC5521157

[61] Gerritsen VB Palagi PM Durinx C: Bioinformatics on a national scale: an example from Switzerland. *Brief Bioinform.* 2019;20(2):361–369. 10.1093/bib/bbx073 29106442 PMC6433736

