# Supplementary material for: The de.NBI / ELIXIR-DE training platform - Bioinformatics training in Germany and across Europe within ELIXIR
Source: F1000Res. 2020 Sep 11;8:ELIXIR-1877. Originally published 2019 Nov 7. [Version 2] doi: 10.12688/f1000research.20244.2 (PMC7607484; doi:10.12688/f1000research.20244.2)
Supplement: Peer review history

# Peer review: Reviewer response for version 2

**DOI:** 10.5256/f1000research.29202.r71234

**Published:** 2020-11-02

**Authors:** Allegra Via, Loredana Le Pera

**Affiliations:** 1 CNR Institute of Biomembranes, Bioenergetics and Molecular Biotechnologies, Bari, Italy; 2 CNR Institute of Molecular Biology and Pathology, Rome, Italy

The written English should be carefully revised throughout the whole paper. ****Here are a few examples:

**Abstract.** The sentence "[...] advertising training courses via TeSS and discussions on the exchange of data for training events essential for quality assessment on both the technical and administrative levels" is not totally sound. Perhaps, "and discussions on the" should be replaced by "and discussing the..."?

**Introduction**: "[...] but the knowledge and skills how to use existing bioinformatics services and tools." --> "[...] but the knowledge and skills **on** how to use existing bioinformatics services and tools."

**Introduction**: I propose that the authors replace "solve these deficiencies" with "fill the existing gaps " or "fill these gaps".

**Introduction**: "[...] handling and analysis of their biological big data [...]". Why only big data? What about data that need statistical and computational skills to be analysed but are not "big"? 

**Introduction**: "The strategic planning of SIG 3 includes following aspects" --> "The strategic planning of SIG 3 includes **the** following aspects"

**Introduction to the de.NBI / ELIXIR-DE training platform**: "Trainer meeting" --> "Trainer meeting **s**"

**Figure 1**: What is the meaning of the circle's size? 

**Figure 1**: Adding the position and name of the main german cities may help non-german readers.

**In The role of de.NBI / ELIXIR-DE ...**: "All de.NBI training course belong to the ELIXIR training program and are available on ELIXIR’s Training Portal, TeSS" --> "All de.NBI training course **s** belong to the ELIXIR training program and are available on **the** ELIXIR’s Training Portal, TeSS"

**In The role of de.NBI / ELIXIR-DE ...**: Check subject-verb consistency in the following sentences: "With the ELIXIR Staff Exchange Program for Galaxy Train-the-Trainer (TtT) events, SIG 3 can exchange knowledge and training skills with the different ELIXIR training communities and aim to build and provide high quality training resources. In association with members from the ELIXIR training network, the Galaxy Training Network and The Carpentries, SIG 3 also contribute to building a full curriculum on the computational analysis of high throughput sequencing data and runs the two corresponding hybrid workshops."

**In The role of de.NBI / ELIXIR-DE ...**: "implementations studies within ELIXIR." --> "implementation studies within ELIXIR."

**In The role of de.NBI / ELIXIR-DE ...: **In my opinion, this entire section should be moved elsewhere, as it doesn't seem to fit in the "Cloud-based training" section. In particular, in the current (revised) version of the paper, its subject has already been partially addressed in the sections "Introduction to ELIXIR-DE" and "Introduction to the de.NBI / ELIXIR-DE training platform", so a possibility could be to integrate its content within these sections.

**Figure 2**: Since user's satisfaction and recommendations are not indicative of course performance, I strongly suggest that the authors modify the legend (e.g. "User's satisfaction and recommendations in each de.NBI service center in the period 2015-2019").

**Future plans and conclusion:**  ... "above ELIXIR average" should be replaced by "equal or superior to ELIXIR average" given that, regarding the recommendation rate, the percentage provided by the ELIXIR Training Metrics Database (up to the end of July 2020) is 89,8%. Therefore, the difference between the ELIXIR-DE value and the ELIXIR value seems not significant.

 In **Future plans and conclusion**: Further --> Furthermore

Is the topic of the opinion article discussed accurately in the context of the current literature?

Partly

Are arguments sufficiently supported by evidence from the published literature?

Yes

Are all factual statements correct and adequately supported by citations?

Yes

Are the conclusions drawn balanced and justified on the basis of the presented arguments?

Partly

Reviewer Expertise:

Bioinformatics, bioinformatics teaching and training, learning processes, training platform coordination

We confirm that we have read this submission and believe that we have an appropriate level of expertise to confirm that it is of an acceptable scientific standard.

# Peer review: Reviewer response for version 1

**DOI:** 10.5256/f1000research.22242.r56339

**Published:** 2019-12-09

**Authors:** Allegra Via, Loredana Le Pera

**Affiliations:** 1 CNR Institute of Biomembranes, Bioenergetics and Molecular Biotechnologies, Bari, Italy; 2 CNR Institute of Molecular Biology and Pathology, Rome, Italy

The paper is a quite detailed description of the research foci and training activities carried out at service centres belonging to the German Network for Bioinformatics Infrastructure (de.NBI).

**General comments:**

**1)** As the paper is presented as a description of the "de.NBI / ELIXIR-DE training platform", the relationship between de.NBI and ELIXIR-DE should be clearly described at the beginning of the paper (instead of being a sub-section of the Cloud section at the end of the paper). Indeed, except for a vague statement in the abstract ("The de.NBI members also run the German node (ELIXIR-DE) within the European ELIXIR network". Who are de.NBI members? What does it mean that they run ELIXIR-DE?), the reader becomes aware of the nature of this relationship only immediately before the conclusions.

 Confusion also arises from the following sentence in the Introduction:

 "The following topics are currently in the focus of SIG 3:

 [...]

1. Integration into the ELIXIR training platform and TeSS
2. Coordination of training events and standards with ELIXIR.

Both "topics" are rather obscure: What does it mean "Integration (of what?) into the ELIXIR training platform and TeSS"? and "Coordination of training events and standards (which standards?) with ELIXIR"? 

 Results are presented as de.NBI activities and the role of ELIXIR-DE is not made explicit throughout the paper. In particular, Germany has become an ELIXR member in 2016, but data presented also include 2015, which further increases reader's confusion about the relationship between de.NBI and ELIXIR-DE.

**2)** Currently, the article is a quite detailed description of research focus and training activities carried out at each service centre belonging to de.NBI and NOT of a "Training Platform". Therefore, it is not clear what the authors mean by "de.NBI / ELIXIR-DE training platform". 

 Ideally, in a Training Platform (TP):

- Courses/events are presented as part of a coherent programme run at different locations/centres, not as an aggregation of courses that each individual centre/institution runs with (apparently) no coordination with other centres/institutions;
- The structure of the TP (including the role of ELIXIR-DE) is described, including who coordinates it and how; who is actively involved in training activities; how many people are involved; what are their roles; how decisions are taken; etc.
- Vision, pedagogical model(s), teaching approaches, goals, future plans should be discussed/explained, as well as how trainers are recruited/selected and how course planning and dissemination is managed; etc...

Based on these considerations, if the authors really mean to present a de.NBI / ELIXIR-DE TP, they should adopt a different perspective, which means try to display courses in a more structured way. For example, grouped by topic and by providing summary information about each type of course. Our suggestion is to use a table with, e.g., the following columns:

- Type of course/course category (e.g. sequencing data analysis).
- Example courses of each category (e.g. microbial sequence data analysis, RNA-Seq and Bisulfite sequencing data analysis in cancer research).
- Brief overview of the content (better if written in the form of general learning outcomes).
- Average number of courses of each type run yearly/monthly/etc.
- Location(s) (service centres/institutes) where courses are delivered.
- Average number of participants.
-  Delivery mode (F2F, e-learning, hackathon, mentoring).
- Etc...

Narrative could be used to describe key features of the platform (as listed above) and of courses. e-learning model(s) also could be better described (synchronous?asynchronous? Blended? etc). A description of service centres and their research foci could be provided very succinctly in Figure 1 or in a separate table (listing, for each centre, the research focus and member institutes) and then leave further details to the literature reference.

**3)** Figure 1 could be made way more informative and clear:

- What is the meaning of the circle's size?
- How should the reader interpret two adjacent circles of the same colour?
- The title of the figure reads: "Training topics and locations of the eight de.NBI service centres in Germany and their connections with existing German and European communities", but:
  - Training topics do not appear in the figure.
  - For clarity, location of main German cities should be shown.
  - The figure shows existing communities and event types but not their connection with service centres.

**Specific comments/questions:**

- What do you mean by "Big Data Problem"? Could you add a reference and explain what characterises it as a "problem"?
- In the abstract, we suggest that you replace "European ELIXIR network" with "European ELIXIR infrastructure".
- What do you mean by "standardisation of training course monitoring"?
- A sentence on what is TeSS should be written before mentioning it in the Introduction.
- p. 4: Re: "Qualification of more trainers." How do you qualify trainers? What is a "qualified trainer"?
- As SIG3 is for "Training & Education", do you also have programs for undergraduates and high school students?
- In the abstract, the overall number of participants is updated to October 2019 ("since 2015, more than 250 training courses were carried out with more than 5,200 participants"), whereas in the paper it is updated to July 2019 ([...] more than 4800 participants (Status July 2019)). This data should be made consistent.
- Regarding performance, authors claim that de.NBI and ELIXIR-DE guarantee standards [...] above ELIXIR average. This statement should be supported by quantitative data.
- p.11: re the sentence: "ELIXIR Germany training coordinators", does Germany have more than one ELIXIR TrC?
- p.11: what is the "general ELIXIR training feedback"? Do you mean Impact study? If so, you should cite the paper on impact currently under revision at PLoS Computational Biology.
- p.11: Re: "new e-learning working group and of the FAIR training working group". You should provide a reference/link to ELIXIR working groups. For example, FAIR training WG: https://github.com/elixir-europe/FAIR-Training.

Is the topic of the opinion article discussed accurately in the context of the current literature?

Partly

Are arguments sufficiently supported by evidence from the published literature?

Yes

Are all factual statements correct and adequately supported by citations?

Yes

Are the conclusions drawn balanced and justified on the basis of the presented arguments?

Partly

Reviewer Expertise:

Bioinformatics, bioinformatics teaching and training, learning processes, training platform coordination

We confirm that we have read this submission and believe that we have an appropriate level of expertise to confirm that it is of an acceptable scientific standard, however we have significant reservations, as outlined above.

# Response

**Published:** 2020-08-28

**Authors:** Daniel Wibberg

**Affiliations:** ELIXIR Germany, Germany

**Reviewer 2: Loredana Le Pera & Allegra Via**

**The paper is a quite detailed description of the research foci and training activities carried out at service centres belonging to the German Network for Bioinformatics Infrastructure (de.NBI).**

**General comments:**

**1) As the paper is presented as a description of the "de.NBI / ELIXIR-DE training platform", the relationship between de.NBI and ELIXIR-DE should be clearly described at the beginning of the paper (instead of being a sub-section of the Cloud section at the end of the paper). Indeed, except for a vague statement in the abstract ("The de.NBI members also run the German node (ELIXIR-DE) within the European ELIXIR network". Who are de.NBI members? What does it mean that they run ELIXIR-DE?), the reader becomes aware of the nature of this relationship only immediately before the conclusions.**

**Confusion also arises from the following sentence in the Introduction:**

**"The following topics are currently in the focus of SIG 3:**

**[...]**

**Integration into the ELIXIR training platform and TeSS**

**Coordination of training events and standards with ELIXIR.**

**Both "topics" are rather obscure: What does it mean "Integration (of what?) into the ELIXIR training platform and TeSS"? and "Coordination of training events and standards (which standards?) with ELIXIR"?**

**Results are presented as de.NBI activities and the role of ELIXIR-DE is not made explicit throughout the paper. In particular, Germany has become an ELIXR member in 2016, but data presented also include 2015, which further increases reader's confusion about the relationship between de.NBI and ELIXIR-DE.**

 Thank you for the comment. We included an additional introduction to the ELIXIR-DE to describe the difference between de.NBI and ELIXIR-DE. Nevertheless, SIG3 is coordinating the training program of both networks resulting in a 100% overlap.

**2) Currently, the article is a quite detailed description of research focus and training activities carried out at each service centre belonging to de.NBI and NOT of a "Training Platform". Therefore, it is not clear what the authors mean by "de.NBI / ELIXIR-DE training platform".**

**Ideally, in a Training Platform (TP):**

**Courses/events are presented as part of a coherent programme run at different locations/centres, not as an aggregation of courses that each individual centre/institution runs with (apparently) no coordination with other centres/institutions;**

**The structure of the TP (including the role of ELIXIR-DE) is described, including who coordinates it and how; who is actively involved in training activities; how many people are involved; what are their roles; how decisions are taken; etc.**

**Vision, pedagogical model(s), teaching approaches, goals, future plans should be discussed/explained, as well as how trainers are recruited/selected and how course planning and dissemination is managed; etc...**

**Based on these considerations, if the authors really mean to present a de.NBI / ELIXIR-DE TP, they should adopt a different perspective, which means try to display courses in a more structured way. For example, grouped by topic and by providing summary information about each type of course. Our suggestion is to use a table with, e.g., the following columns:**

**Type of course/course category (e.g. sequencing data analysis).**

**Example courses of each category (e.g. microbial sequence data analysis, RNA-Seq and Bisulfite sequencing data analysis in cancer research).**

**Brief overview of the content (better if written in the form of general learning outcomes).**

**Average number of courses of each type run yearly/monthly/etc.**

**Location(s) (service centres/institutes) where courses are delivered.**

**Average number of participants.**

**Delivery mode (F2F, e-learning, hackathon, mentoring).**

**Etc...**

**Narrative could be used to describe key features of the platform (as listed above) and of courses. e-learning model(s) also could be better described (synchronous?asynchronous? Blended? etc). A description of service centres and their research foci could be provided very succinctly in Figure 1 or in a separate table (listing, for each centre, the research focus and member institutes) and then leave further details to the literature reference.**

 Thank you for the comment. In general, our courses are on an annual basis. Therefore, we provide in 80 courses almost 80 different types of courses. Therefore, such a table would be too large to present in the paper. The service centers have unique topics in de.NBI / ELIXIR-DE. Therefore, there is almost no topic overlap in the training courses between the different service centers. That is the reason, why we choose this strategy to present the training program. To describe the training platform in detail, we included an additional part introducing the training platform.

**3) Figure 1 could be made way more informative and clear:**

**What is the meaning of the circle's size?**

**How should the reader interpret two adjacent circles of the same colour?**

**The title of the figure reads: "Training topics and locations of the eight de.NBI service centres in Germany and their connections with existing German and European communities", but:**

**Training topics do not appear in the figure.**

**For clarity, location of main German cities should be shown.**

**The figure shows existing communities and event types but not their connection with service centres.**

 Thank you for the comment. The circle size is now explained in the figure legend. The service centers represent the training topics. As explained before, each service center has its topic and there is almost no overlap with another center.

**Specific comments/questions:**

**What do you mean by "Big Data Problem"? Could you add a reference and explain what characterises it as a "problem"?**

 Was added

**In the abstract, we suggest that you replace "European ELIXIR network" with "European ELIXIR infrastructure".**

 Was replaced

**What do you mean by "standardisation of training course monitoring"?**

 We developed a standardized form that must be filled out after every course and user survey based the ELIXIR survey questions.

**A sentence on what is TeSS should be written before mentioning it in the Introduction.**

 A sentence on TeSS was added.

**p. 4: Re: "Qualification of more trainers." How do you qualify trainers? What is a "qualified trainer"?**

 We plan to organize more Train-the-Trainer and Software Carpentry Instructor course. In addition, a trainer community meeting is planned.

**As SIG3 is for "Training & Education", do you also have programs for undergraduates and high school students?**

 No, we don’t have a special program for undergraduates and high school students. About 15% of all participants are undergraduates and we support a summer school for high school students.

**In the abstract, the overall number of participants is updated to October 2019 ("since 2015, more than 250 training courses were carried out with more than 5,200 participants"), whereas in the paper it is updated to July 2019 ([...] more than 4800 participants (Status July 2019)). This data should be made consistent.**

 Was updated to July 2020.

**Regarding performance, authors claim that de.NBI and ELIXIR-DE guarantee standards [...] above ELIXIR average. This statement should be supported by quantitative data.**

 Quantitative data was added.

**p.11: re the sentence: "ELIXIR Germany training coordinators", does Germany have more than one ELIXIR TrC?**

 Yes, one chair and two deputies

**p.11: what is the "general ELIXIR training feedback"? Do you mean Impact study? If so, you should cite the paper on impact currently under revision at PLoS Computational Biology.**

 Citation was added

**p.11: Re: "new e-learning working group and of the FAIR training working group". You should provide a reference/link to ELIXIR working groups. For example, FAIR training WG:** **https://github.com/elixir-europe/FAIR-Training** **.**

 Reference was added

# Peer review: Reviewer response for version 1

**DOI:** 10.5256/f1000research.22242.r56336

**Published:** 2019-11-14

**Authors:** Roland Krause

**Affiliations:** 1 Luxembourg Centre for Systems Biomedicine, University of Luxembourg, Esch-sur-Alzette, Luxembourg

The review of the training activities in the de.NBI network focuses on the description of the courses delivered as well as the contributing centers. Partially owing to the complex set-up of the centers it is a bit short on evaluation of what has worked and what was learned in the process. Including more experiences - courses that were not oversubscribed and those that were would improve the manuscript significantly. I cannot believe that no other lessons were learned after delivering ~200 courses. The discussion of lessons learned  is vary valuable to course providers in other infrastructures (such as me). I acknowledge that such critical reviews might not find universal support across the many people involved in the training activities in de.NBI.

 The structure of the overview can be improved by shortening the sections devoted to the individual centers and instead discussing items that are taught across all centers, such as basic statistics courses. 

 The contribution from the individual centers are too long and frequently include vague or obvious statements (existence of an HPC facility, the online announcement of course and requirements). A stronger editing mandate would be advised. 

 Software developed in a particular center for which training is provided should only be mentioned in the section that discusses the training. There is not usually need to repeat them multiple times in the text.

 Figures:

- Figure 1. The composition of the individual centers by cities is difficult even for someone acquainted with the geography of Germany. There is space to include the names of the centers, which could then receive less coverage in the text.
- Figure 2. Has no content, would recommend to delete. The geographic spread of de.NBI nodes using AAI provides no insight beyond the names.
- Figure 3. The course title is repeated in caption and should be removed from the graphics. Altogether the whole figure could be represented on a easy to comprehend 3x4 table with a higher data to ink ratio. It would be more interesting to see the number of open seats per course and/or a resolution, maybe at the level of quarters.
- Figure 4. Pie charts. 4B is only repeating information already included in full in the text. Again, it would be more interesting to see the distribution of the courses at a different level, e.g. to see if some courses are doing very well or whether the numbers are similarly overall. It's not important to learn which courses exactly under perform but more what the course provider thought about the cause and what was done to rectify it. 

Is the topic of the opinion article discussed accurately in the context of the current literature?

Yes

Are arguments sufficiently supported by evidence from the published literature?

Yes

Are all factual statements correct and adequately supported by citations?

Yes

Are the conclusions drawn balanced and justified on the basis of the presented arguments?

Yes

Reviewer Expertise:

Bioinformatics, training

I confirm that I have read this submission and believe that I have an appropriate level of expertise to confirm that it is of an acceptable scientific standard.

# Response

**Published:** 2020-08-28

**Authors:** Daniel Wibberg

**Affiliations:** ELIXIR Germany, Germany

**Reviewer 1: Roland Krause**

**The review of the training activities in the de.NBI network focuses on the description of the courses delivered as well as the contributing centers. Partially owing to the complex set-up of the centers it is a bit short on evaluation of what has worked and what was learned in the process. Including more experiences - courses that were not oversubscribed and those that were would improve the manuscript significantly. I cannot believe that no other lessons were learned after delivering ~200 courses. The discussion of lessons learned is vary valuable to course providers in other infrastructures (such as me). I acknowledge that such critical reviews might not find universal support across the many people involved in the training activities in de.NBI.**

 Thank you for the comment. Well, almost all courses are oversubscribed and in 5 years of de.NBI, we had to cancel only four courses because of a low amount of participants. Our learned lessons are

- Not to organize courses in the summer or Christmas holidays
- Provide a high diversity of training course topics
- Focusing of beginners, but don’t forget advance and expert users
- More day courses are more interesting than one day courses

In our point of view, these points are not so interesting for the paper. We want to focus the paper on the training activities of de.NBI / ELIXIR-DE

**The structure of the overview can be improved by shortening the sections devoted to the individual centers and instead discussing items that are taught across all centers, such as basic statistics courses.**

 Thank you for the comment. We shortened the service center chapters. Based on the topics of each service center, there is almost no overlap in the training program of the service centers. There are a few collaboration within service centers in training course, e.g. the summer school or the annual Nanopore workshop, but normally one service center provide one specific type of training.

**The contribution from the individual centers are too long and frequently include vague or obvious statements (existence of an HPC facility, the online announcement of course and requirements). A stronger editing mandate would be advised.**

 Thank you for the comment. The chapters were edited and shortened.

**Software developed in a particular center for which training is provided should only be mentioned in the section that discusses the training. There is not usually need to repeat them multiple times in the text.**

 Thank you for the comment. Sometimes, the software needs to be mentioned to underline the research focus of the center. Repeats were reduced.

**Figures:**

**Figure 1. The composition of the individual centers by cities is difficult even for someone acquainted with the geography of Germany. There is space to include the names of the centers, which could then receive less coverage in the text.**

 The names of the service centers are mentioned on the left side of the figure. The color underlines the belonging to a service center

**Figure 2. Has no content, would recommend to delete. The geographic spread of de.NBI nodes using AAI provides no insight beyond the names.**

 Thank you for the comment. The figure was deleted.

**Figure 3. The course title is repeated in caption and should be removed from the graphics. Altogether the whole figure could be represented on a easy to comprehend 3x4 table with a higher data to ink ratio. It would be more interesting to see the number of open seats per course and/or a resolution, maybe at the level of quarters.**

 Thank you for the comment. The figure was replaced by a table (s. table 1).

**Figure 4. Pie charts. 4B is only repeating information already included in full in the text. Again, it would be more interesting to see the distribution of the courses at a different level, e.g. to see if some courses are doing very well or whether the numbers are similarly overall. It's not important to learn which courses exactly under perform but more what the course provider thought about the cause and what was done to rectify it.**

 Thank you for the comment. We included here a new figure showing the similar performance of all course providers (s. Figure 2).
